# Ethyl α-d-sorboside monohydrate

**DOI:** 10.1107/S2414314626008047

**Published:** 2026-08-07

**Authors:** Tomoko Takashita, Yoji Horii, Kei Takeshita, Tomohiko Ishii

**Affiliations:** a Graduate School of Science for Creative Emergence Kagawa University, 2217-20 Hayashi-cho Takamatsu Kagawa 761-0396 Japan; b FUSHIMI Pharmaceutical Co., Ltd., 307 Minatomachi, Marugame, Kagawa 763-8605, Japan; Katholieke Universiteit Leuven, Belgium

**Keywords:** crystal structure, hydrogen bonding, rare sugar, ethyl sorboside, monohydrate

## Abstract

The title compound, C_8_H_16_O_6_·H_2_O, was prepared by Fischer glycosyl­ation of d-sorbose with ethanol. Colorless block-shaped single crystals suitable for single-crystal X-ray diffraction were obtained. The title compound crystallizes in the ortho­rhom­bic space group *P*2_1_2_1_2_1_, with one sorboside mol­ecule and one water mol­ecule in the asymmetric unit. The sorboside mol­ecule adopts an α-pyran­ose form with a ^5^*C*_2_ chair conformation. In the crystal, the sorboside and water mol­ecules are linked by O—H⋯O hydrogen bonds, forming an extended hydrogen-bonded network.

## Abstract 2

The title compound, ethyl α-d-sorboside monohydrate, was prepared by Fischer glycosyl­ation of d-sorbose with ethanol. The crystal structure contains one water mol­ecule per sorboside mol­ecule, and the components are linked by O—H⋯O hydrogen bonds.

## Graphical abstract

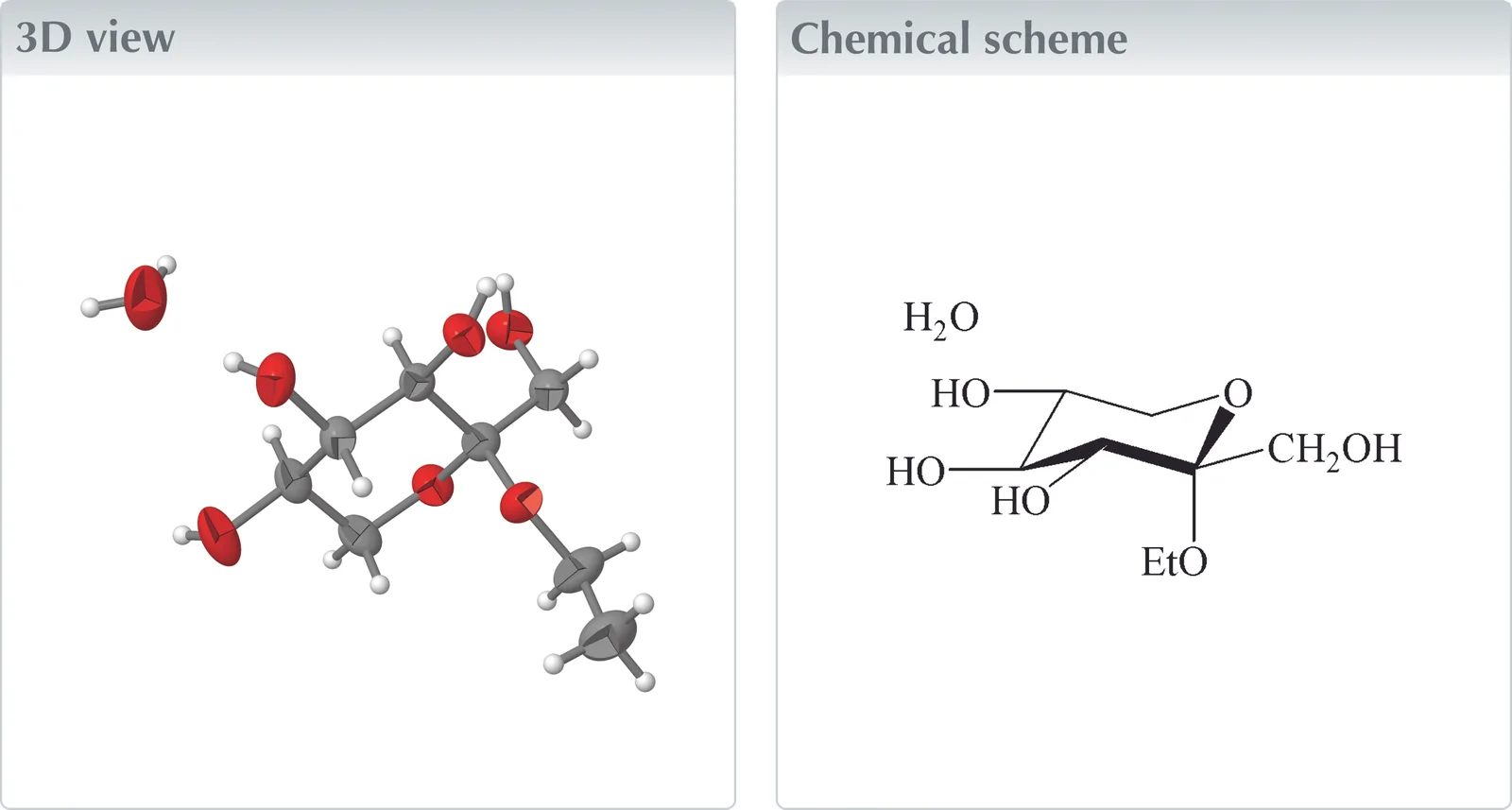

## Structure description

Rare sugars are monosaccharides and their derivatives that occur only in limited qu­anti­ties in nature (Izumori, 2002 ▸). d-Sorbose is a rare ketohexose, and its alkyl glycosides provide useful examples for examining how substitution at the anomeric centre affects mol­ecular conformation, hydration and crystal packing. The title compound, ethyl α-d-sorboside monohydrate, is an ethyl glycoside of d-sorbose in which the anomeric hy­droxy group at C2 is replaced by an eth­oxy group. In the present study, its single-crystal structure was determined and compared with the previously reported anhydrous structure of ethyl α-l-sorboside (Nagayama *et al.*, 2020 ▸).

**Figure 1:**
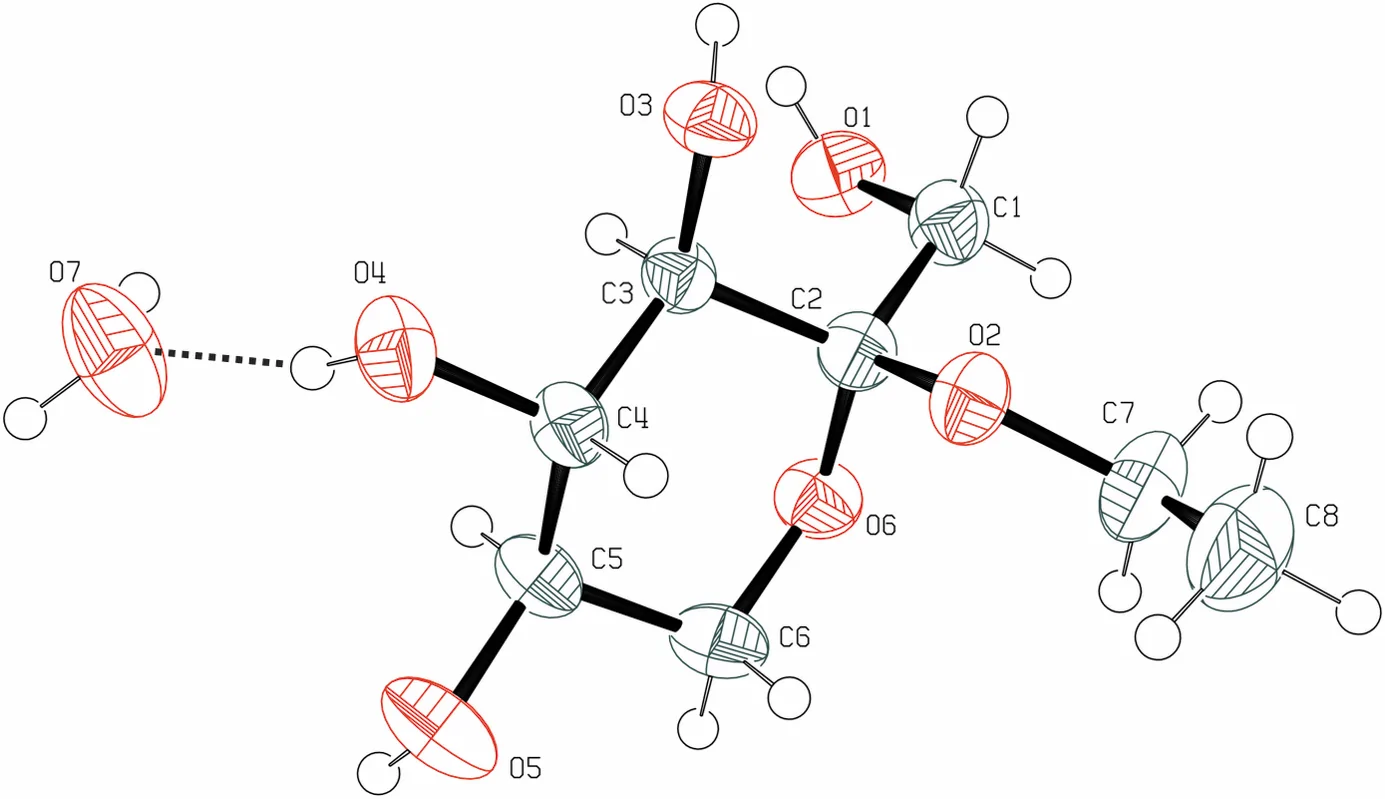
The mol­ecular structure of the asymmetric unit of the title compound, showing the atom-labeling scheme. Displacement ellipsoids are drawn at the 50% probability level. Hydrogen atoms are shown as spheres of arbitrary radius. The O4—H4⋯O7 hydrogen bond between the sorboside and water mol­ecules is shown as a dashed line.

The title compound crystallizes in the ortho­rhom­bic space group *P*2_1_2_1_2_1_. The asymmetric unit contains one ethyl α-d-sorboside mol­ecule and one water mol­ecule. The sorboside mol­ecule adopts an α-pyran­ose form with a ^5^*C*_2_ chair conformation and the eth­oxy substituent at the anomeric C2 atom occupying an axial position (Fig. 1 ▸). The stereogenic centres C2, C3, C4 and C5 have *S*, *R*, *S* and *R* configurations, respectively. The C2—O2—C7—C8 torsion angle is −173.8 (2)°.

The anhydrous crystal structure of ethyl α-l-sorboside (CSD refcode EJAKAE; Nagayama *et al.*, 2020 ▸) also belongs to space group *P*2_1_2_1_2_1_, with a unit-cell volume of 940.63 (19) Å^3^ and *Z* = 4. The unit-cell volume of the present monohydrate is therefore 151.35 Å^3^ (16.1%) larger. This difference is consistent with the inclusion of four water mol­ecules of crystallization per unit cell, together with the resulting change in crystal packing. After inversion of the l-sorboside mol­ecule, least-squares fitting of all 14 non-hydrogen atoms to the corresponding atoms of the present d-sorboside mol­ecule gave an r.m.s. deviation of 0.441 Å The principal conformational difference is the orientation of the hydroxymethyl group at C1, as indicated by the O1—C1—C2—C3 torsion angles of −1257.1 (3)° in the title monohydrate and −153.2 (2)° in the inverted EJAKAE molecule. By contrast, the sorbopyranose ring conformations and the *anti* conformations of the ethoxy groups are closely similar.

**Figure 2:**
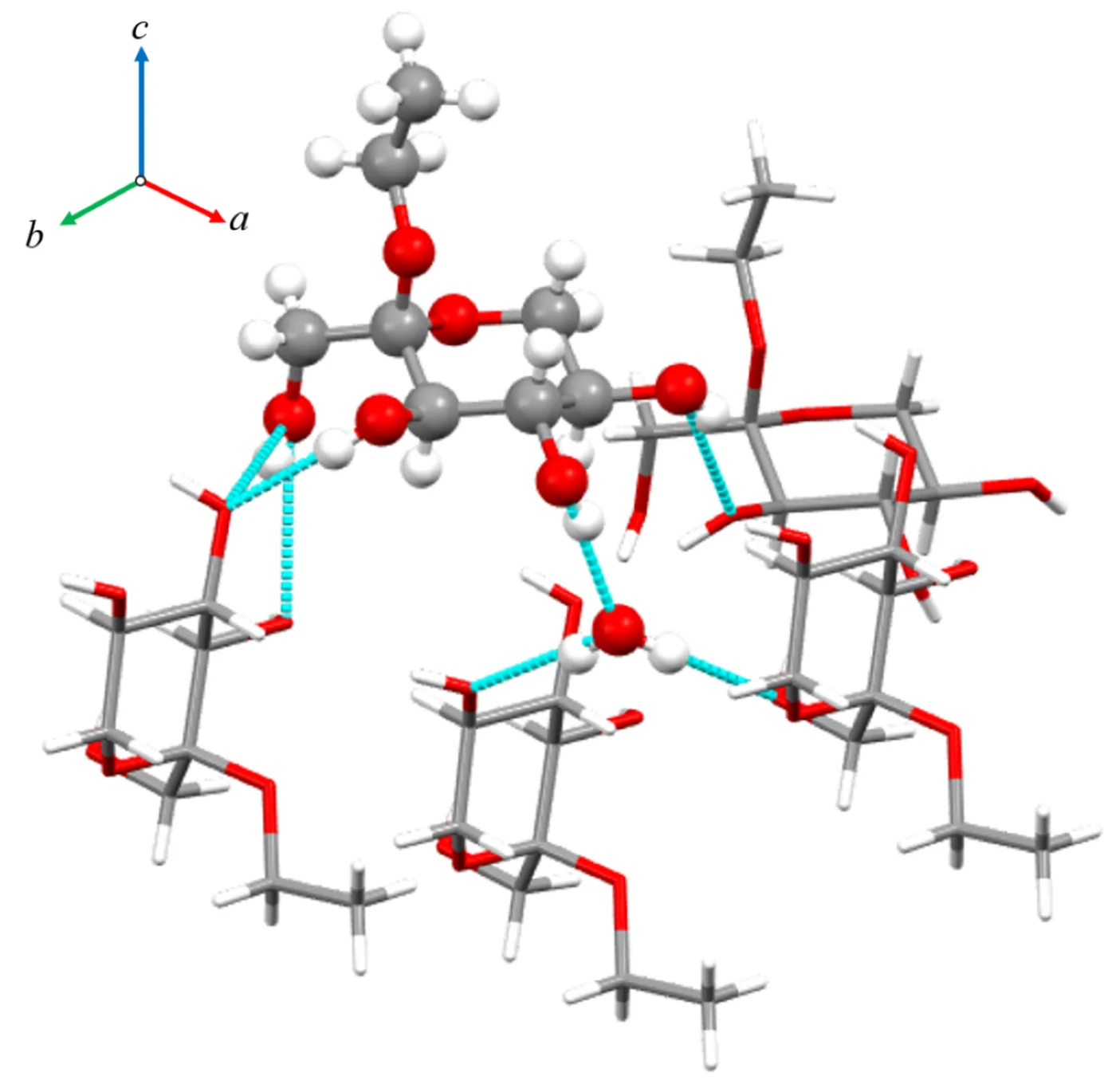
A portion of the crystal packing of the title compound, shown in an oblique view with the unit-cell directions indicated. The central sorboside mol­ecule and the water mol­ecule are shown using a ball-and-stick representation, whereas the surrounding sorboside mol­ecules are shown using a capped-stick representation. The seven crystallographically distinct O—H⋯O hydrogen bonds listed in Table 1 ▸ are shown as cyan dashed lines.

In the crystal, the sorboside and water mol­ecules are connected by the O1—H1⋯O3, O1—H1⋯O4, O3—H3⋯O4, O4—H4⋯O7, O5—H5⋯O3, O7—H7C⋯O5 and O7—H7D⋯O1 hydrogen bonds listed in Table 1 ▸. The O1—H1 group participates in a bifurcated hydrogen bond, with the O3 and O4 atoms acting as acceptors. The donor⋯acceptor distances range from 2.618 (3) to 3.018 (2) Å. Taken together, the seven O—H⋯O hydrogen bonds generate a two-dimensional hydrogen-bonded network extending parallel to the (001) plane. The crystal packing and all seven hydrogen bonds listed in Table 1 ▸ are shown in Fig. 2 ▸.

## Synthesis and crystallization

Ethyl α-d-sorboside monohydrate was prepared by Fischer glycosyl­ation of d-sorbose with ethanol. Because the reaction produced a mixture of isomeric products, including α- and β-anomers and furan­ose forms, the reaction mixture was separated by ion-exchange chromatography. Fractions containing the desired product were combined and concentrated to give a syrup, which was allowed to stand at room temperature. Colorless block-shaped single crystals suitable for single-crystal X-ray diffraction were obtained. The absolute configuration was assigned on the basis of the known configuration of the d-sorbose starting material and the synthetic route.

## Refinement

Crystal data, data collection and structure refinement details are summarized in Table 2 ▸. The Flack parameter supports the absolute configuration expected from the use of d-sorbose as the starting material.

## Supplementary Material

Crystal structure: contains datablock(s) I. DOI: 10.1107/S2414314626008047/vm4080sup1.cif

Structure factors: contains datablock(s) I. DOI: 10.1107/S2414314626008047/vm4080Isup3.hkl

Point-by-point response to the Co-editors comments, detailing the revisions made to the manuscript, Table 2, and Figures 1 and 2 (manuscript vm4080). DOI: 10.1107/S2414314626008047/vm4080sup3.docx

CCDC reference: 2577733

Additional supporting information:  crystallographic information; 3D view; checkCIF report

## Figures and Tables

**Table 1:** Hydrogen-bond geometry (Å, °)

*D*—H⋯*A*	*D*—H	H⋯*A*	*D*⋯*A*	*D*—H⋯*A*
O1—H1⋯O3^i^	0.82	2.36	3.018 (2)	137
O1—H1⋯O4^i^	0.82	2.25	2.975 (3)	148
O3—H3⋯O4^i^	0.82	1.93	2.741 (2)	167
O4—H4⋯O7	0.82	1.82	2.618 (3)	165
O5—H5⋯O3^ii^	0.82	2.08	2.842 (3)	154
O7—H7*C*⋯O5^iii^	0.85	1.90	2.738 (3)	168
O7—H7*D*⋯O1^iv^	0.85	2.09	2.909 (3)	163

Symmetry codes: (i) 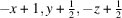; (ii) 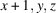; (iii) 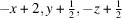; (iv) 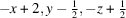.

**Table 2:** Experimental details

Crystal data	
Chemical formula	C_8_H_16_O_6_·H_2_O
*M*_r_	226.22
Crystal system, space group	Orthorhombic, *P*2_1_2_1_2_1_
Temperature (K)	296
*a*, *b*, *c* (Å)	6.6457 (2), 7.5616 (2), 21.7300 (6)
*V* (Å^3^)	1091.98 (5)
*Z*	4
Radiation type	Cu *K*α
μ (mm^−1^)	1.05
Crystal size (mm)	0.1 × 0.1 × 0.1

Data collection	
Diffractometer	Rigaku R-AXIS RAPID
Absorption correction	Multi-scan (*ABSCOR*; Rigaku, 1995 ▸)
*T*_min_, *T*_max_	0.702, 1.000
No. of measured, independent and observed [*I* > 2σ(*I*)] reflections	12255, 1969, 1706
*R*_int_	0.054
(sin θ/λ)_max_ (Å^−1^)	0.602

Refinement	
*R*[*F*^2^ > 2σ(*F*^2^)], *wR*(*F*^2^), *S*	0.031, 0.076, 1.02
No. of reflections	1969
No. of parameters	144
H-atom treatment	H-atom parameters constrained
Δρ_max_, Δρ_min_ (e Å^−3^)	0.15, −0.13
Absolute structure	Flack *x* determined using 626 quotients [(*I*^+^)−(*I*^−^)]/[(*I*^+^)+(*I*^−^)] (Parsons *et al.*, 2013 ▸)
Absolute structure parameter	−0.04 (11)

Computer programs: *RAPID-AUTO* (Rigaku, 2009 ▸), *SHELXT2018/2* (Sheldrick, 2015*a* ▸), *SHELXL2018/3* (Sheldrick, 2015*b* ▸) and *OLEX2* (Dolomanov *et al.*, 2009 ▸).

## full crystallographic data

### Ethyl α-D-sorboside monohydrate Crystal data

**Table:**

C_8_H_16_O_6_·H_2_O	*D*_x_ = 1.376 Mg m^−^^3^
*M**_r_* = 226.22	Cu *K*α radiation, λ = 1.5418 Å
Orthorhombic, *P*2_1_2_1_2_1_	Cell parameters from 11105 reflections
*a* = 6.6457 (2) Å	θ = 4.1–68.1°
*b* = 7.5616 (2) Å	µ = 1.05 mm^−^^1^
*c* = 21.7300 (6) Å	*T* = 296 K
*V* = 1091.98 (5) Å^3^	Block, clear light colourless
*Z* = 4	0.1 × 0.1 × 0.1 mm
*F*(000) = 488	

### Ethyl α-D-sorboside monohydrate Data collection

**Table:**

Rigaku R-AXIS RAPID diffractometer	1706 reflections with *I* > 2σ(*I*)
ω scans	*R*_int_ = 0.054
Absorption correction: multi-scan (ABSCOR; Rigaku, 1995)	θ_max_ = 68.1°, θ_min_ = 4.1°
*T*_min_ = 0.702, *T*_max_ = 1.000	*h* = −7→7
12255 measured reflections	*k* = −9→9
1969 independent reflections	*l* = −26→25

### Ethyl α-D-sorboside monohydrate Refinement

**Table:**

Refinement on *F*^2^	Hydrogen site location: mixed
Least-squares matrix: full	H-atom parameters constrained
*R*[*F*^2^ > 2σ(*F*^2^)] = 0.031	*w* = 1/[σ^2^(*F*_o_^2^) + (0.0327*P*)^2^ + 0.1352*P*] where *P* = (*F*_o_^2^ + 2*F*_c_^2^)/3
*wR*(*F*^2^) = 0.076	(Δ/σ)_max_ < 0.001
*S* = 1.02	Δρ_max_ = 0.15 e Å^−^^3^
1969 reflections	Δρ_min_ = −0.13 e Å^−^^3^
144 parameters	Absolute structure: Flack *x* determined using 626 quotients [(*I*^+^)-(*I*^-^)]/[(*I*^+^)+(*I*^-^)] (Parsons *et al.*, 2013)
0 restraints	Absolute structure parameter: −0.04 (11)
Primary atom site location: dual	

### Ethyl α-D-sorboside monohydrate Special details

**Table:**

Geometry. All esds (except the esd in the dihedral angle between two l.s. planes) are estimated using the full covariance matrix. The cell esds are taken into account individually in the estimation of esds in distances, angles and torsion angles; correlations between esds in cell parameters are only used when they are defined by crystal symmetry. An approximate (isotropic) treatment of cell esds is used for estimating esds involving l.s. planes.
Refinement. The structure was solved using SHELXT (Sheldrick, 2015*a*) and refined against F^2^ by full-matrix least-squares methods using *SHELXL* (Sheldrick, 2015*b*). All non-hydrogen atoms were refined anisotropically. Hydrogen atoms were placed in calculated positions and refined using constrained models. The final refinement gave *R*_1_ = 0.0311 for reflections with I > 2σ(I) and w*R*_2_ = 0.0759 for all data. The maximum and minimum residual electron densities were 0.15 and -0.13 e Å^-3^, respectively.

### Ethyl α-D-sorboside monohydrate Fractional atomic coordinates and isotropic or equivalent isotropic displacement parameters (Å^2^)

**Table:**

	*x*	*y*	*z*	*U*_iso_*/*U*_eq_	
O1	0.5536 (3)	0.9834 (2)	0.34665 (8)	0.0487 (5)	
H1	0.502283	0.981931	0.312334	0.073*	
O2	0.4803 (2)	0.5405 (2)	0.41294 (7)	0.0409 (4)	
O3	0.4025 (2)	0.5562 (2)	0.28922 (7)	0.0392 (4)	
H3	0.348364	0.643371	0.274062	0.059*	
O4	0.7416 (3)	0.3772 (2)	0.24941 (7)	0.0443 (5)	
H4	0.840422	0.417639	0.231721	0.066*	
O5	1.0724 (3)	0.3576 (2)	0.33542 (10)	0.0572 (5)	
H5	1.187162	0.394017	0.329875	0.086*	
O6	0.7715 (3)	0.7138 (2)	0.40269 (7)	0.0409 (4)	
C1	0.4643 (4)	0.8509 (3)	0.38368 (11)	0.0418 (6)	
H1A	0.326162	0.833089	0.370681	0.050*	
H1B	0.462452	0.890214	0.426162	0.050*	
C2	0.5773 (4)	0.6760 (3)	0.37952 (10)	0.0350 (5)	
C3	0.5945 (3)	0.6048 (3)	0.31342 (10)	0.0314 (5)	
H3A	0.651070	0.698141	0.287380	0.038*	
C4	0.7331 (4)	0.4455 (3)	0.31061 (10)	0.0350 (5)	
H4A	0.679010	0.353462	0.337700	0.042*	
C5	0.9361 (4)	0.5024 (3)	0.33518 (11)	0.0390 (6)	
H5A	0.990207	0.597101	0.309203	0.047*	
C6	0.9110 (4)	0.5700 (4)	0.40010 (11)	0.0455 (6)	
H6A	0.863529	0.474734	0.426218	0.055*	
H6B	1.040394	0.608870	0.415706	0.055*	
C7	0.4558 (5)	0.5640 (4)	0.47787 (11)	0.0590 (8)	
H7A	0.365283	0.661921	0.485898	0.071*	
H7B	0.584626	0.590126	0.496772	0.071*	
C8	0.3727 (6)	0.4001 (5)	0.50366 (15)	0.0775 (11)	
H8A	0.243423	0.377111	0.485613	0.116*	
H8B	0.358345	0.412393	0.547409	0.116*	
H8C	0.461880	0.303491	0.494816	0.116*	
O7	1.0151 (3)	0.5050 (3)	0.17499 (12)	0.0711 (7)	
H7C	1.005187	0.615896	0.169385	0.107*	
H7D	1.134246	0.479972	0.163735	0.107*	

### Ethyl α-D-sorboside monohydrate Atomic displacement parameters (Å^2^)

**Table:**

	*U*^11^	*U*^22^	*U*^33^	*U*^12^	*U*^13^	*U*^23^
O1	0.0558 (12)	0.0388 (10)	0.0513 (11)	−0.0007 (9)	−0.0042 (9)	0.0038 (8)
O2	0.0448 (10)	0.0422 (9)	0.0355 (8)	−0.0050 (8)	0.0050 (7)	0.0031 (8)
O3	0.0291 (9)	0.0433 (10)	0.0452 (10)	0.0004 (9)	−0.0072 (7)	−0.0025 (8)
O4	0.0376 (10)	0.0476 (10)	0.0477 (10)	−0.0057 (9)	0.0075 (8)	−0.0148 (8)
O5	0.0296 (10)	0.0428 (10)	0.0992 (15)	0.0029 (9)	0.0004 (10)	−0.0055 (10)
O6	0.0369 (9)	0.0417 (10)	0.0440 (9)	−0.0032 (8)	−0.0075 (8)	−0.0067 (7)
C1	0.0453 (15)	0.0396 (14)	0.0405 (13)	0.0025 (12)	0.0016 (11)	−0.0047 (11)
C2	0.0331 (13)	0.0361 (13)	0.0359 (12)	−0.0046 (11)	−0.0009 (11)	−0.0009 (10)
C3	0.0276 (12)	0.0334 (12)	0.0332 (11)	−0.0029 (11)	0.0009 (9)	−0.0002 (9)
C4	0.0311 (12)	0.0350 (13)	0.0389 (13)	−0.0015 (12)	0.0031 (10)	−0.0042 (11)
C5	0.0275 (13)	0.0353 (14)	0.0543 (14)	−0.0002 (10)	0.0006 (11)	−0.0022 (11)
C6	0.0329 (13)	0.0487 (15)	0.0549 (15)	0.0018 (13)	−0.0119 (11)	−0.0002 (13)
C7	0.070 (2)	0.072 (2)	0.0348 (13)	−0.0081 (19)	0.0069 (13)	0.0024 (14)
C8	0.091 (3)	0.082 (2)	0.060 (2)	0.002 (2)	0.0174 (18)	0.0237 (18)
O7	0.0619 (14)	0.0485 (13)	0.1028 (17)	0.0016 (10)	0.0380 (12)	0.0090 (11)

### Ethyl α-D-sorboside monohydrate Geometric parameters (Å, º)

**Table:**

O1—H1	0.8200	C3—H3A	0.9800
O1—C1	1.415 (3)	C3—C4	1.517 (3)
O2—C2	1.412 (3)	C4—H4A	0.9800
O2—C7	1.431 (3)	C4—C5	1.514 (3)
O3—H3	0.8200	C5—H5A	0.9800
O3—C3	1.428 (3)	C5—C6	1.510 (3)
O4—H4	0.8200	C6—H6A	0.9700
O4—C4	1.428 (3)	C6—H6B	0.9700
O5—H5	0.8200	C7—H7A	0.9700
O5—C5	1.421 (3)	C7—H7B	0.9700
O6—C2	1.414 (3)	C7—C8	1.468 (4)
O6—C6	1.430 (3)	C8—H8A	0.9600
C1—H1A	0.9700	C8—H8B	0.9600
C1—H1B	0.9700	C8—H8C	0.9600
C1—C2	1.524 (3)	O7—H7C	0.8501
C2—C3	1.538 (3)	O7—H7D	0.8498
			
C1—O1—H1	109.5	C5—C4—C3	107.55 (19)
C2—O2—C7	118.0 (2)	C5—C4—H4A	108.5
C3—O3—H3	109.5	O5—C5—C4	110.50 (18)
C4—O4—H4	109.5	O5—C5—H5A	109.4
C5—O5—H5	109.5	O5—C5—C6	109.2 (2)
C2—O6—C6	115.07 (18)	C4—C5—H5A	109.4
O1—C1—H1A	109.2	C6—C5—C4	109.10 (19)
O1—C1—H1B	109.2	C6—C5—H5A	109.4
O1—C1—C2	112.0 (2)	O6—C6—C5	111.47 (19)
H1A—C1—H1B	107.9	O6—C6—H6A	109.3
C2—C1—H1A	109.2	O6—C6—H6B	109.3
C2—C1—H1B	109.2	C5—C6—H6A	109.3
O2—C2—O6	112.37 (18)	C5—C6—H6B	109.3
O2—C2—C1	111.97 (18)	H6A—C6—H6B	108.0
O2—C2—C3	105.08 (18)	O2—C7—H7A	110.0
O6—C2—C1	104.65 (19)	O2—C7—H7B	110.0
O6—C2—C3	109.62 (19)	O2—C7—C8	108.3 (3)
C1—C2—C3	113.31 (19)	H7A—C7—H7B	108.4
O3—C3—C2	111.58 (18)	C8—C7—H7A	110.0
O3—C3—H3A	108.4	C8—C7—H7B	110.0
O3—C3—C4	108.88 (18)	C7—C8—H8A	109.5
C2—C3—H3A	108.4	C7—C8—H8B	109.5
C4—C3—C2	111.12 (18)	C7—C8—H8C	109.5
C4—C3—H3A	108.4	H8A—C8—H8B	109.5
O4—C4—C3	110.43 (18)	H8A—C8—H8C	109.5
O4—C4—H4A	108.5	H8B—C8—H8C	109.5
O4—C4—C5	113.33 (19)	H7C—O7—H7D	104.5
C3—C4—H4A	108.5		
			
O1—C1—C2—O2	−175.77 (18)	C2—O2—C7—C8	−173.8 (2)
O1—C1—C2—O6	62.2 (2)	C2—O6—C6—C5	57.3 (3)
O1—C1—C2—C3	−57.1 (3)	C2—C3—C4—O4	177.90 (18)
O2—C2—C3—O3	56.1 (2)	C2—C3—C4—C5	−58.0 (2)
O2—C2—C3—C4	−65.6 (2)	C3—C4—C5—O5	178.40 (18)
O3—C3—C4—O4	54.6 (2)	C3—C4—C5—C6	58.4 (2)
O3—C3—C4—C5	178.73 (18)	C4—C5—C6—O6	−57.9 (3)
O4—C4—C5—O5	−59.3 (3)	C6—O6—C2—O2	61.8 (2)
O4—C4—C5—C6	−179.3 (2)	C6—O6—C2—C1	−176.52 (19)
O5—C5—C6—O6	−178.70 (19)	C6—O6—C2—C3	−54.7 (2)
O6—C2—C3—O3	177.04 (18)	C7—O2—C2—O6	56.5 (3)
O6—C2—C3—C4	55.3 (2)	C7—O2—C2—C1	−61.0 (3)
C1—C2—C3—O3	−66.5 (2)	C7—O2—C2—C3	175.6 (2)
C1—C2—C3—C4	171.8 (2)		

### Ethyl α-D-sorboside monohydrate Hydrogen-bond geometry (Å, º)

**Table:**

*D*—H···*A*	*D*—H	H···*A*	*D*···*A*	*D*—H···*A*
O1—H1···O3^i^	0.82	2.36	3.018 (2)	137
O1—H1···O4^i^	0.82	2.25	2.975 (3)	148
O3—H3···O4^i^	0.82	1.93	2.741 (2)	167
O4—H4···O7	0.82	1.82	2.618 (3)	165
O5—H5···O3^ii^	0.82	2.08	2.842 (3)	154
O7—H7*C*···O5^iii^	0.85	1.90	2.738 (3)	168
O7—H7*D*···O1^iv^	0.85	2.09	2.909 (3)	163

Symmetry codes: (i) −*x*+1, *y*+1/2, −*z*+1/2; (ii) *x*+1, *y*, *z*; (iii) −*x*+2, *y*+1/2, −*z*+1/2; (iv) −*x*+2, *y*−1/2, −*z*+1/2.
