# Supplementary material for: Ethyl α-d-sorboside monohydrate
Source: IUCrdata. 2026 Aug 7;11(Pt 8):x260804. doi: 10.1107/S2414314626008047 (PMC13548378; doi:10.1107/S2414314626008047)
Supplement: Supplementary file 1 [file x-11-x260804-sup3.docx]

Response to Co-editor Professor L. Van Meervelt

Title: Ethyl α-*D*-sorboside monohydrate

Manuscript ID: vm4080

CCDC deposition number: 2577733

Dear Professor Van Meervelt,

We sincerely thank you for your careful assessment of our data article and for your helpful comments. We have revised the manuscript, Table 2, Figure 1 and Figure 2 in accordance with your suggestions. Our point-by-point responses are provided below.

The crystallographic data for the title compound have been deposited with the

Cambridge Crystallographic Data Centre under CCDC deposition number 2577733.

**Comment 1:**

Line 23: add also axial position of -OEt substituent

**Response:**

The axial orientation of the ethoxy substituent has been added to the structural description. The relevant sentence has been revised as follows:

“The sorboside molecule adopts an α-pyranose form with a ^5^*C*_2_ chair conformation, in which the ethoxy substituent at C2 occupies an axial position (Figure 1).”

**Comment 2:**

Line 25: add CCDC refcode EJAKAE for this structure – you can also calculate the rms deviation for fitting both structures

**Response:**

The CSD refcode EJAKAE has been added for the previously reported anhydrous crystal structure of ethyl α-*L*-sorboside. A least-squares overlay of the sorboside molecules in the present monohydrate structure and the previously reported anhydrous structure was also performed. After inversion of one

structure to account for their opposite absolute configurations, the fitting gave an r.m.s. deviation of 0.441 Å. The corresponding discussion has been added to the manuscript.

**Comment 3:**

Line 28 and Table 2: Platon shows three additional H-bonds which are also worthwhile to mention – O7-H7D…O1, O1-H1…O3 and O1-H1…O4 (and show in Fig. 2).

**Response:**

The three additional hydrogen bonds, O7—H7D···O1, O1—H1···O3 and O1—H1···O4, have been added to Table 2 and to the structural description. The O1—H1 group participates in a bifurcated hydrogen bond, with O3 and O4 acting as acceptors. Table 2 now contains seven crystallographically distinct O—H···O hydrogen bonds. All seven hydrogen bonds are shown as cyan dashed lines in the revised Figure 2.

**Comment 4:**

Line 30: check if “parallel to the (001) plane” is still valid (with additional H-bonds)

**Response:**

The dimensionality and orientation of the hydrogen-bonded network were re-examined after inclusion of the three additional hydrogen bonds. These interactions do not generate connectivity between adjacent layers along the *c*-axis. The structure therefore still forms a two-dimensional hydrogen-bonded network extending parallel to the (001) plane. The corresponding sentence has been revised to clarify that this description is based on all seven hydrogen bonds.

**Comment 5:**

Figure 1: use larger atom labels and move labels to avoid overlap with the atom.

**Response:**

Figure 1 has been replaced with a revised ORTEP drawing. The atom labels have been enlarged and repositioned to avoid overlap with the atoms, displacement ellipsoids and bonds. The O4—H4···O7 hydrogen bond between the sorboside and lattice water molecules is shown as a dashed line, and the figure caption has been revised accordingly.

**Comment 6:**

Figure 2: also add the unit cell or unit cell directions + additional H-bonds (see 3.).

**Response:**

Figure 2 has been revised to include the a-, b- and c-axis directions. The three additional hydrogen bonds identified in Comment 3 have also been added, and all seven crystallographically distinct O—H···O hydrogen bonds listed in Table 2 are now shown as cyan dashed lines. The Figure 2 caption has been revised accordingly.

We hope that these revisions have satisfactorily addressed all of the comments and that the revised data article is now suitable for publication in IUCrData.

Yours sincerely,

Tomoko Takashita

Graduate School of Science for Creative Emergence, Kagawa University, Japan
